# Developing and Testing Digital Ethical Reflection in Long-Term Care: Nurses’ Experiences

**DOI:** 10.1177/23779608221150725

**Published:** 2023-01-12

**Authors:** Lena Jakobsen, Rose Mari Olsen, Berit Støre Brinchmann, Siri Andreassen Devik

**Affiliations:** 1 Faculty of Nursing and Health Sciences, 1786 Nord University, Bodø, Norway; 2 Centre for Care Research Mid-Norway, Faculty of Nursing and Health Sciences, 1786 Nord University, Namsos, Norway; 3 Faculty of Nursing and Health Sciences, 1786 Nord University, Norway and Nordland Regional University, Bodø, Norway

**Keywords:** digital ethics support, nurses, ethics, reflection, ethical challenges, mixed methods

## Abstract

**Introduction:**

Nurses working in municipal long-term care face ethical challenges that can lead to moral distress and discomfort for the nurse and affect the quality of patient care. Tools and methods that contribute to increased ethical awareness and support for nurses dealing with moral issues are lacking. Technological innovations may be suitable for ethics work, but little research has been conducted on how such solutions could be designed or their potential benefit. Therefore, this study contributes knowledge about the development and testing of a digital tool for ethics support among nurses.

**Objective:**

To investigate how digital ethical reflection can support ethics work among nurses working in long-term care.

**Methods:**

A digital ethical reflection tool was designed and tested in nursing homes and home nursing care in collaboration with two Norwegian municipalities. The study used sequential explanatory mixed-methods design. Over a 6-week period, at the end of each shift, nurses digitally reported the ethical challenges they had experienced. Their responses and experiences were described using descriptive statistics. Additionally, focus group interviews were conducted and analyzed using reflexive thematic analysis (TA).

**Results:**

During the study period, 17 nurses reported a total of 223 registrations, with 24.8% stating that they had been in an ethically difficult situation. The digital reporting was perceived as practically applicable and helped to increase nurses’ awareness of morally charged situations. The value of the registrations was found to depend on manager participation and the application of the obtained information. The participating nurses become aware that they lacked an arena for meaningful dialogue with and recognition from their manager.

**Conclusions:**

Information obtained through digital reflection can form the basis for ethical reflections at the departmental level. Digital reflection has the potential to become a tool for managers in their support for employees facing ethical challenges when providing long-term care.

## Introduction

The use of digital solutions for ethical reflection is a new approach to ethics support. Although previous studies have examined digital solutions for ethics education (Chao et al., 2017; Johnston & Mok, 2015; Manninen et al., 2020), they have focused on teaching ethics online rather than on overcoming perceived ethical challenges in a clinical context. In addition, digital tools for stress management have been tested in mental health care settings (Hwang & Jo, 2019), but to our knowledge, no studies have been conducted on the processing of moral distress by health care professionals. The use of digital solutions is expected to improve the efficiency and availability of services (Ahtinen et al., 2013). Developing new approaches to helping health care workers deal with ethical challenges is of vital importance both nationally and globally (Rainer et al., 2018). This study responds to the need for an effective and feasible digital solution for supporting ethical reflection among primary care nurses working in long-term care.

### Literature Review

In Norway, long-term care is a tax-financed municipal responsibility provided through services in nursing homes and home nursing care services, with the latter accounting for the largest proportion of services for all patients living at home who need advanced nursing care (Kattouw & Wiig, 2018; Meld.St.15 (2017–2018)). The patient group increasingly consists of elderly people with complex conditions and needs that challenge both capacity and quality of the services (Gautun et al., 2022). Good quality care therefore largely depends on nurses’ ability to make complex, ethical decisions in their practice (Heggestad & Førde, 2020). Ethical challenges and dilemmas are everyday experiences for nurses working in nursing homes and home care services (Hopia et al., 2016; Suhonen et al., 2018). An aging population and short hospital stays, together with limited public funding and a lack of qualified staff, put pressure on the nurses who are responsible for providing care to patients in these settings (Fjørtoft et al., 2021; Leknes et al., 2018). Nurses often feel that they cannot do the “right thing” when practicing nursing and struggle with discrepancies between their professional and personal values, which in turn can lead to moral distress (Haahr et al., 2019; Koskenvuori et al., 2018).

Morally charged situations can lead to stress and feelings of powerlessness, which have an increasingly negative effect on health care workers both personally and professionally (Cohen & Erickson, 2006). The effects of moral distress on nurses include burnout (Austin et al., 2017; Scanlan & Still, 2019), intent to leave the profession (Jameton, 2013; Scanlan & Still, 2019; Wan et al., 2020), lower work satisfaction (Poikkeus et al., 2020) and dissatisfaction with their organization's ethical climate (Epstein et al., 2019). The negative effects of moral distress are apparent in all health care disciplines (Houston et al., 2013) and negatively impact the quality of patient care (Goethals et al., 2010; Jokwiro et al., 2022).

Reduced moral sensitivity is one potential consequence of inappropriate management of moral distress, which can in turn make nurses indifferent to the quality of patient care (Amiri et al., 2018). Another term often used for this situation is “compassion fatigue”, defined as the negative cost of caring that occurs when health care professionals are unable to care for patients as much as they should (Figley, 1995). The ethical competence of nurses has a strong impact on the quality of care provided (Koskenvuori et al., 2018; Papastavrou et al., 2014).

When nurses are given the opportunity to handle moral challenges successfully, they may experience personal growth (Hanna, 2004) and enhanced moral resilience (Rushton et al., 2017). Bjerkvik and Hilli's (2019) study on reflective writing by undergraduate nursing students demonstrated that writing down one's experiences can help initiate the reflective process. Kuiper and Pesut (2004) point out that structured guidelines are helpful for writers, particularly newly educated nurses, and that such self-regulated learning can enhance clinical reasoning skills.

Interventions are increasingly used in health care to relieve the moral distress experienced by nurses (Epstein et al., 2019; Moss et al., 2016; Perni, 2017; Ulrich et al., 2010). Various approaches have been developed to support ethical awareness and foster decision-making skills among health care personnel (Rasoal et al., 2017). The most commonly available ethics support services described in the literature are clinical ethics committees, moral case deliberation, ethics rounds, ethics discussion groups, and ethics reflection groups. Deliberation, discussion, and reflection groups are based on the idea that health care personnel needs to discuss and reflect critically on the ethical issues that they face in their daily clinical practice (Rasoal et al., 2017).

Ethics reflection groups are the most widely used method of ethics support for nurses working in long-term care in Norway. They were popularized by a national ethics project involving 243 municipalities undertaken from 2007 to 2015 (Magelssen et al., 2016b). Despite positive results, including increased competence, better handling of ethical challenges, and improved cooperation skills, ethics work such as reflection groups has since stagnated or disappeared in 40% of the municipalities (Magelssen et al., 2016a). Barriers to continued ethics work include organizational, structural, and financial obstacles, as well as a lack of ethical competence (Bruun et al., 2019; Karlsen et al., 2018). It is challenging to prioritize time for ethical reflection and facilitate meaningful discussions of the practical issues faced by employees (Devik et al., 2020; Munkeby et al., 2021). Moreover, nurse managers require specialized tools and methods to strengthen long-term care employees’ ethical competence (Devik et al., 2020; Stolt et al., 2017).

In recent years, there has been optimism about the potential for digital solutions to promote health and coping with working life (Ebert et al., 2014; Hwang & Jo, 2019; Muuraiskangas et al., 2016). The objectives of digital technology initiatives have mainly been training and stress reduction (Ebert et al., 2014; Hwang & Jo, 2019; Valencia, 2020), and the results have been mixed, revealing barriers at both the individual and organizational levels.

The development of a digital solution for ethical reflection took place in collaboration with two municipalities in Mid-Norway and the Centre for Development of Institutional and Home Care Services (CDIH) in the county of Trøndelag from 2018 to 2019. The research conducted for this development is the first of its kind. The present study is part of a larger project, led by the first author, which is developing methods for systematic ethics work among nurses.

This study aimed to investigate how digital ethical reflection can support ethics work among nurses working in long-term care by investigating the following research questions: (a) What information about ethical challenges can be gained when nurses reflect digitally? (b) How do nurses experience digital ethical reflection? (c) What are the benefits and limitations of digital ethical reflection in the context of providing ethics support for nurses working in long-term care?

## Method

### Design

**Figure 1.**
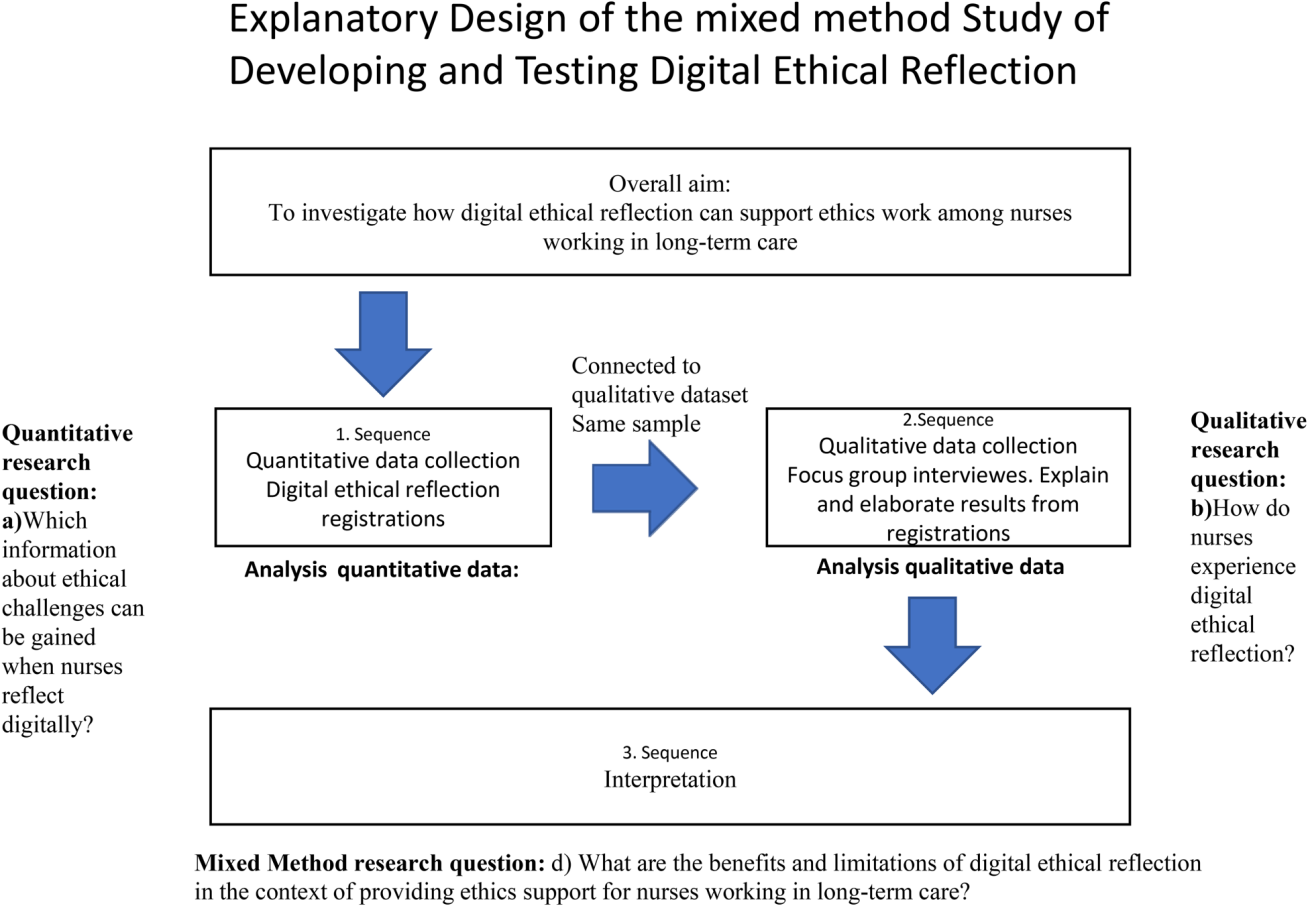
The sequential explanatory mixed-methods process of the project.

Because we sought practical knowledge, we chose a pragmatic method (Glasgow, 2013). A sequential explanatory mixed-methods design was applied (Figure 1) (Creswell, 2021). During the testing of the ethical reflection, participants quantitatively registered the moral dilemmas that they encountered during the shifts worked in a given period. In the subsequent qualitative part of the test, focus group interviews were conducted with the same participants. The qualitative part aimed to explore the use of digital ethical reflection as a tool for ethics support, including its applicability, benefits, and limitations. The qualitative method of focus group interviews was used to obtain further explanation and contextual analysis of the quantitative findings, as well as contribute to the future development of the digital tool (Tashakkori & Creswell, 2007).

### The Development of Digital Ethical Reflection

The project group consisted of two nurse managers, representing nursing homes and home care services, in two medium-sized municipalities (between 15,000 and 25,000 inhabitants) in mid-Norway, a professional advisor from CDIH, and the research team. The project group had regular meetings and workshops both before start-up and during the testing period. The development of the digital ethical reflection was motivated by the managers’ difficulties with supporting their employees’ handling of everyday ethics. The managers were particularly concerned about the management of moral distress among their employees. They believed that their nursing staff needed opportunities to express ethical challenges experienced at work. At the same time, they suspected that their employees experienced substantial instances of moral distress of which the managers were unaware. The digital ethical reflection tool was launched to enable employees to effectively identify and report ethical challenges and provide managers with information about the “ethical temperature” of the department. This information is potentially useful for managers when applied systematically to ethics work—for example, as a starting point in ethics reflection groups.

**Figure 2.**
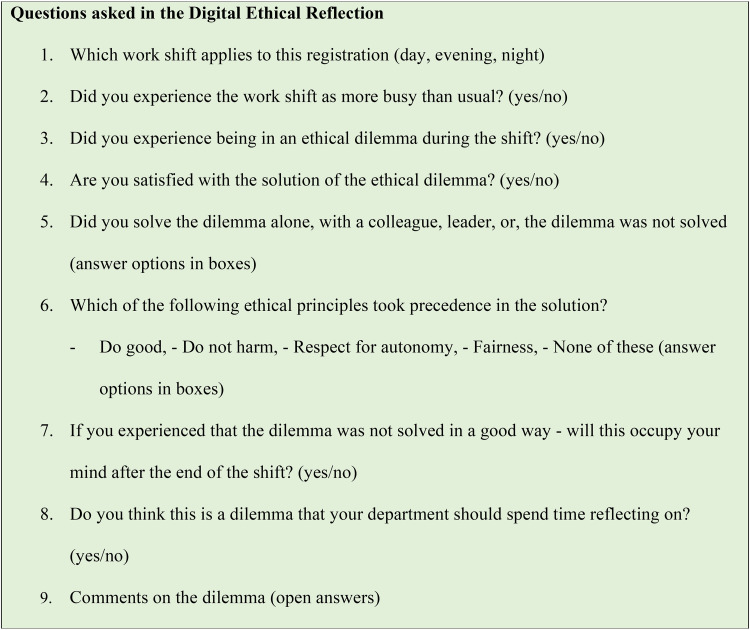
Questions asked in the digital ethical reflection.

According to Moula and Sandin (2015), an ethical tool is a practical method intended to help the user(s) improve their ethical deliberations to reach ethically informed judgements and decisions. Determining the quality of an ethical tool is challenging, but one possible metric of quality is how accessible the tool is for the users (Moula & Sandin, 2015). The project group, therefore, decided that testing should take place with a limited number of employees working in both nursing homes and home nursing care. We implemented the simple solution of distributing a nine-item questionnaire that could be completed within 5 to 10 min. A digital link to the digital ethical reflection was provided to employees to complete via email or short message service (SMS). The information we sought was limited to context, ethical dilemma, solution, and need for follow-up (Figure 2). The questions were developed by researchers in collaboration with two nurse managers and were inspired by previous research in the field and the theory of ethical principles for solving moral dilemmas (Beauchamp & Childress, 2009; Beauchamp & Childress, 2019; Edwards, 2009; Johns, 2017).

Although interpretations of ethical concepts vary among theorists, researchers, and practitioners (Schofield et al., 2021), we chose to use the term “ethical dilemma” and classified the values at stake based on the four biomedical ethical principles (Beauchamp & Childress, 2009; Beauchamp & Childress, 2019). Our rationale was that these principles are familiar to practitioners and would allow us to obtain categorized answers. In addition, previous research shows that practitioners tend to use the terms “dilemma” and “challenge” interchangeably (Munkeby et al., 2021). Moral distress can arise as a psychological response regardless of whether the difficulty is defined as a dilemma, conflict, or uncertainty (Campbell et al., 2018; Fourie, 2013).

As a first step in the development of digital support for ethics work, we focused on nurses’ experiences.

### Setting and Participants

The recruitment of participants took place with the help of two nurse managers in the municipalities that participated in the study, who contacted health care workers in the service areas they were responsible for. Information about the project and invitation to participate was conveyed both in writing and orally. In addition to a willingness to participate, the inclusion criteria were that the participants had to work as health care workers, either in nursing homes or home nursing care, in at least a 50% position. The last author established contact with interested participants to provide in-depth information and obtain consent. The participants, 17 nurses in total, represented a mixed skill set: eight registered nurses (RNs), five enrolled nurses (ENs), and four assistant nurses (ANs). Ten of the participants worked in home nursing care and seven worked in nursing homes. They were all women and their ages ranged between 29 and 63 years (median = 46 years). Among the 17 nurses who participated in the quantitative registration, 14 of them agreed to participate in focus group interviews conducted from January to February 2019. The groups were homogeneous in the sense that two consisted of participants from home nursing care (Groups 1 and 2) and the third of participants from nursing homes (Group 3). An overview of the participants in the focus groups is shown in Table 1.

**Table 1.** Overview of Participants in Focus Groups.

Profession	Persons	Age^a^	Group 1 (home care)	Group 2 (home care)	Group 3 (nursing home)
Assistant nurse (AN)	3	57	1	1	1
Enrolled nurse (EN)	4	56	1	2	1
Registered nurse (RN)	7	42	3	2	2

^a^ Age in median.

### Data Collection

Data were collected using digital registrations and focus group interviews.

#### The Digital Ethical Reflection

The digital ethical reflection, conducted with an online survey, consisted of nine questions (Figure 2) which the nurses were invited to answer after each work shift. The nurses answered the questions digitally through a link provided via SMS or email, using either a mobile phone or computer. They responded by selecting the answer option that suited them best. One of the questions provided an opportunity to submit comments. The nurses were instructed to make one registration per dilemma. During the project period, 223 responses were registered covering various work shifts: day, evening, and night.

#### Focus Group Interviews

Focus group interviews were performed as described by Morgan (1996) and conducted physically in one meeting room at one of the nursing homes and one meeting room in a municipal health care center. Because the participants worked in different nursing homes and geographical zones in home nursing care, the meeting rooms that were used were perceived as neutral ground for most of the participants. The interviews were conducted by a moderator and audio recordings were made. The interviews were based on an interview guide that facilitated discussions about the usability and usefulness of the digital reflection. The following questions were asked: How did you experience the practical implementation of this form of ethical reflection? How did you perceive the relevance of the questions? Did you find this form of ethical reflection useful in any way? Is this a form of ethical reflection that you would like to continue with? Follow-up questions were asked to obtain more comprehensive answers and examples and to foster discussion among the participants. The interviews lasted between 70 and 80 min. Audio recordings were transcribed verbatim.

### Analysis

The two data sets were analyzed separately, with the statistical analysis of the digital registrations preceding the reflexive thematic analysis (TA) of the focus group interviews.

#### Statistical Analysis

The registrations from the digital ethical reflection were analyzed using SPSS software version 28.0 for Windows (IBM Corporation, Armonk, NY, USA). Descriptive statistics were reported as frequencies and percentages. A chi-squared test was conducted to compare the proportions of ethical challenges. A *p*-value of less than .05 was considered statistically significant.

#### Reflexive TA

The transcripts from the focus group interviews were analyzed using reflexive TA (Braun & Clarke, 2019). Using reflexive TA means that influences from the researchers’ subjective interpretations are acknowledged as a resource for the analysis process (Braun & Clarke, 2020), and was chosen as best suited to capture people's experiences, thoughts and meanings when trying to understand how digital ethical reflection can support ethics work in this test. The authorship team consisted of RNs with prior experience of working in both nursing homes and home nursing care, as well as experience of being in nurse management positions. One of the researchers has experience of working in hospital settings. An initial reading was carried out, followed by a rereading of the interview transcripts by the first and last author. The first reading enabled us to recognize various patterns of shared meanings in the text. Meaningful units were then identified and encoded. Initial subthemes were processed from the codes and finally thermalized. The analytical process was carried out by moving back and forth between inductive and deductive analysis. The analysis was initiated by the first author, but codes, subthemes, and themes were discussed and explored by all the researchers jointly during the analytical process. Their different views contributed to deeper insights.

### Ethical Considerations

The participants in the study were informed both in writing and orally about the purpose of the study, confidentiality, and the possibility of withdrawing consent. Written consent was obtained from all participants. The study was approved by the Norwegian Centre for Research Data (project number 59444). The digital responses were collected anonymously without any personally identifying information such as name, phone number, or email address.

## Findings

Since we used a sequential explanatory mixed-methods sequential design, the results from each phase are presented separately and the integration of the findings is conveyed as a unified account in the discussion (Cresswell & Plano Clark, 2011).

### Quantitative Results

A total of 223 registrations were submitted in 6 weeks by 17 nurses, most of them during day shifts (Table 2). More than half of the registrations indicated that the ward was as busy as usual, while 23.5% of the shifts were described as busier than usual. The experience of an ethical dilemma during the shift was reported in 24.8% of the cases. Ethical dilemmas were reported significantly more often by nurses who described their shifts as busier than usual (46.0%) than by those who described their shifts as less busy or as busy as usual (18.1%) (*p* < 0.001), and by nurses who had worked evening or night shifts (35.6%) than by those who had worked day shifts (20.5%) (*p* = 0.023). Half of the ethical issues were resolved together with colleagues. In one-third of the cases, the dilemmas were managed by the nurse themselves. Very few were dealt with by consulting the nurse manager. Most (86.4%) of the nurses were satisfied with how their dilemma was resolved, but 13.7% did not find a solution.

**Table 2.** Registrations From the Digital Ethical Reflection (N = 223).

Response	N	%
*Type of shift*		
Day	156	70.3
Evening	61	27.5
Night	5	2.3
N = 222 (missing: 1)		
*Bustle*		
Less than usual	22	10.3
As usual	141	66.2
More than usual	50	23.5
N = 213 (missing: 10)		
*Experienced ethical dilemma*		
Yes	52	24.8
No	158	75.2
N = 210 (missing: 13)		
*How was the dilemma resolved?*		
By myself	16	31.4
With a colleague	26	51.0
With the manager	2	3.9
Was not solved	7	13.7
N = 51		
*Satisfied with the solution?*		
Yes	38	86.4
No	6	13.6
N = 44		
*Dominant ethical principle*		
Beneficence	10	25.0
Nonmaleficence	5	12.5
Autonomy	23	57.5
Justice	2	5.0
N = 40 (missing: 4)		
*Occupy my mind after the shift*		
Yes	17	33.3
No	34	66.7
N = 51		
*Requires ethical reflection in the ward*		
Yes	32	64.0
No	18	36.0
N = 50 (missing: 1)		

In more than half (57.5%) of the registrations, the principle of “respect for autonomy” was emphasized most in finding a solution to the dilemma. The principle of “doing good” was also emphasized often while “justice” was reported less frequently (4.2%) and “no harm” was registered in only 2.2% of cases. Most nurses (66.7%) stated that the ethical challenges they experienced did not occupy their thoughts after their shift. Many of the registrations showed that the nurses wanted the department to devote time to reflection on perceived ethical issues (64%).

### Qualitative Findings

The reflexive TA resulted in four themes: practical applicability of the digital ethical reflection, increased attention to morally charged situations, depending on the managers’ attention, and missing an arena for meaningful dialogue. An overview of subthemes and themes is shown in Table 3.

**Table 3.** Overview of Subthemes and Themes.

Subthemes	Themes
Not time-consuming	*Practical applicability of the digital ethical reflection*
Comprehensible usage of terms	
Available on various platforms	
Catalyst for reflection	*Increased attention to morally charged situations*
Awareness of own attitudes	
Reminded of the ethical aspects	
Need to communicate their thoughts	*Depending on the managers’ attention*
Gives the manager insight	
Digital registrations cannot reflect the real world	*Missing an arena for meaningful dialogue*
Aware of the need for in-depth discussions	
Need to deliberate with co-workers	

#### Practical Applicability of the Digital Ethical Reflection

Accessibility, in terms of time, place, and digital login method, was an important issue. Some participants used work mobiles or tablets, while others chose to use their own mobile phones or PC. Some answered the questions at home and some at work. It was most important that the questions were available when the participants felt that they had time to think through their workday and complete the registration. For some, it was important that the manager openly encouraged the nurses to complete the registration. Some also received compensation for the time spent on the registration, which was perceived as a sign that management thought the project was important.

“You have to prioritise a little and at the end of a working day it becomes a bit like… quickly done… you just have to hurry on. If time has been allocated, as we have now – half an hour per week, then time would not have been so inhibiting in a way then.” RN—Group 3

Several of the participants chose to complete their registration at home in peace and quiet. The questions were perceived as easy to understand and posed no problems for the participants. Many of the participants described how difficult it was for both them and their colleagues to find the motivation to participate in something new and change their routine. They described an internal sense of resistance and that they felt lost in the face of “something new”. One of the participants said that there was not enough pressure on the nurses from the organization to make changes to improve nursing care or contribute to quality improvement in the department. Several participants found it difficult not only to talk in plenary meetings but especially to address the unethical behavior of colleagues. Using the digital reflection made it easier to express their opinions and to shed light on the wrongdoings of other nurses:

“It's good if it can become a type of anonymous temperature measurement. Then you don’t have to sit and agree with the ‘gang’, when you in fact don’t agree with things that are said or done.” RN—Group 3

They felt that their voices could be heard too, rather than only those who spoke the loudest and most frequently in the groups. The anonymity provided by the digital solution felt reassuring.

#### Increased Attention to Morally Charged Situations

Many participants said that they had increased awareness of how they had acted in specific situations after completing the registration. Some felt that the questions forced them to think twice and consider which situations might have been ethically challenging, while others did not feel that they had experienced any ethically challenging situations or dilemmas during the workday:

“I think I’m so run-in, I had to think about it… ‘Gosh, have I been in an ethical dilemma?’ And when I found out that I hadn’t been in an ethical dilemma, I thought it must be wrong too – because I’ve probably run into something without thinking about it… So, I really think I’ve become more aware of it, so that's awesome.” RN—Group 2

For some, the registration served as a reminder to think retrospectively about how they had acted in a particular situation. It also allowed them to assess situations in which they should have sought help from a colleague or the manager to solve an ethical challenge. One participant stated that writing about the challenges felt good:

“I thought it was good to write a little in the comment textbox… to sort and clarify what the challenge was. It was okay to do that at the end of the day. You take it with you… and make it a little bit more visible for yourself. It was really good to write it down. You go through a lot of ethical issues all the time, but you might not think much about them right there and then and that there's a dilemma… for example, shared decision-making. It's a dilemma all the time.” AN—Group 1

Some respondents stated that using the digital reflection helped them clarify their own needs and reflect on a deeper level. However, the complexity of the ethical challenges faced in daily practice was outside the scope of a fixed questionnaire, according to many respondents.

#### Depending on the Managers’ Attention

For the majority of the nurses, it was important that someone noted and paid attention to their registrations in the digital ethical reflection. The nurses spent time and effort completing their registrations and reporting how they had experienced their workdays and any ethical challenges they had faced. If the manager did not pay attention to the registrations, they felt it was meaningless. The nurses wished to be heard and seen, and the manager was often unavailable when the nurses needed to discuss ethical challenges:

“If you knew this was going to the manager, you might have put a little more effort into it and described the situations you were in as well… I think that it would be a good idea. Then you could feel it was going to a place where things could be picked up.” RN—Group 2

For many participants, completing the registrations was also about professional development. They saw the digital ethical reflection as an opportunity to work actively toward professional enhancement and collaboration in the department. This presupposed that “someone”, preferably the manager, would take note of the feedback and take responsibility for working through the challenges with the staff:

“The digital reflection can be a tool that indicates that now we are in a work period where we are a little too busy. It will be a reminder or warning signal for all of us that we should calm down and look at what we are doing and why.” RN—Group 2

Prioritizing between tasks and patients and conducting assessments of patients’ capacities were often mentioned by the participants as ethical challenges not covered by the questions in the digital reflection. According to the nurses, the quality of care deteriorated when their workload was perceived as too heavy. Some participants stated that the efficiency demanded by their organization was affecting patient care, making the nurses feel powerless.

#### Missing an Arena for Meaningful Dialogue

The period during which the nurses completed the registrations made them aware that they lacked an outlet for ethical reflection. Many participants expressed a desire to discuss ethical challenges at the time when they arose. They would find a corner in which they could have an ad hoc conversation when the pressure became overwhelming:

“We work very purposefully all the time and there is little time for conversations and reflection. We’ve probably lost some venues there… then you end up talking in places where you’re not supposed to talk, so to speak. It's not good I think… to stand in the sluice room to talk… in terms of confidentiality and other things… there will be whispers around the corners… but it's because we need to solve things.” RN—Group 2

The arenas previously provided to discuss ethical challenges, such as oral reports, had been removed from several workplaces and replaced with “silent reports” (a form of written documentation) about the patients. Most of the participants desired a forum for more in-depth discussion on ethical issues but also expressed the need for ethics work in all settings of everyday life:

“I have really always missed the ethics discussions and wished that things were put more into a system, and that we have tools to carry it out so that it becomes something proper of it. If not, it’ll just be regular conversations.” RN—Group 3

In addition to increased awareness of the lack of an arena for in-depth ethical reflection, many participants also became more aware of the need for social connection with one another. The ability to talk and support each other was considered more important than doing solitary reflections:

“Ethical reflection… what is that? We’re not that familiar with it or may not even have it in our vocabulary. I believe that everyone in a working environment must take responsibility for making sure that there is open space for ethical reflection… Because I’ve felt it a few times… We need space to talk about what's happened to us during the day… Call it what you want. We work with people. We are human beings, and we need human contact.” RN—Group 2

Several participants pointed out that many meeting opportunities for employees had been removed by the management, increasing their feelings of loneliness in their jobs.

## Discussion

The aim of this study was to investigate how digital ethical reflection can support ethics work among nurses in long-term care. Our findings show that a digital approach has both advantages and limitations. The use of digital reporting seemed to increase nurses’ ethical sensitivity but also made them more aware of their need for in-depth reflections and discussions. Most evident was the need for acknowledgment and involvement from their managers.

### The Content of Ethical Challenges

While previous research has suggested that nurses face daily ethical challenges (Hopia et al., 2016; Raines, 2000), the digital reporting of such challenges was surprisingly low in our sample. However, the infrequent reports of ethical dilemmas contradict the experiences shared by the participants in the focus group interviews. The interviews showed that the nurses often had trouble recalling situations after their shifts, despite their belief that they continuously faced ethical difficulties. Moreover, the nurses viewed everyday ethics as diverse and not always easy to categorize according to defined principles. This may be due either to the biomedical ethical principles being too narrow and thus not always fitting the complex situations faced by nurses (Walker, 1993), or to nurses lacking the time or experience to engage in ethical reasoning in their daily work (Milliken & Grace, 2015).

In line with the findings of previous research (Heggestad et al., 2021; Rykkje et al., 2021), the principle of autonomy was the subject of the dilemma most often faced by nurses. The proportion of patients with cognitive impairment is high in long-term care and questions of shared decision making and autonomy are challenges often faced when caring for these patients. Ethical dilemmas related to prioritizing between patients and tasks and dealing with heavy workloads were discussed in the interviews but did not appear in the digital reporting, where higher scores for the principle of “justice” might have been expected. However, significantly more dilemmas were recorded on shifts that were characterized as “busier than usual”. This accords with other research in the field showing that perceived discrepancies between workload and resources are not only practical but also ethically difficult for nurses (Haahr et al., 2019; Munkeby et al., 2021). The digital reflection also showed that dilemmas were more often recorded on evening and night shifts than on day shifts. Staffing is normally lower in the evenings and at night, which can lead to an increased sense of busyness for nurses on shift while also having fewer colleagues to interact and confer with.

### Experiences of Ethics Support

Having the opportunity to discuss ethical difficulties with colleagues was highlighted as an important theme in the focus groups. The digital responses also showed that cooperation with colleagues was essential for finding solutions to dilemmas. The nurses were clear that their need for discussion and reflection was not fully covered by the digital registration, but that it could nevertheless help identify and bring attention to problems. The use of the digital registration presupposes that there is a recipient who will respond to the answers. The attention and involvement of managers were called for in all focus groups. Meeting this need for attention is vital for helping the nurses to alleviate stressful feelings (Edwards et al., 2013; Honkavuo & Lindström, 2012). The digital registrations would only be meaningful if they were looked into further and made the subject of oral reflection in the department. This was also evident in the digital responses, where a majority indicated that they wanted ethical issues to be dealt with at the departmental level. Having the opportunity to discuss norms and values with colleagues can contribute to a sense of reflective and moral conformity and build an ethical climate in the department. Morality is interpersonal, collaborative, and continuously redeveloped in social contexts in which everyone is mutually responsible and accountable to one another (Walker, 2007). A number of studies have shown that ethical reflection with a bottom-up approach can increase the moral awareness and accountability of health care workers, as well as provide support and foster readiness for action (Hem et al., 2018; Magelssen et al., 2016a; Rasoal et al., 2017). At the same time, such reflections require competence in both ethics and management. The nurses in our study stated that it was difficult to distinguish daily conversation from ethical reflection. Although it is natural to address colleagues and the department community when ethical challenges arise, this can also be a problematic practice. Colleagues can have a strong impact on each other; un-reflective conformity on moral issues among nurses can become an obstacle to ethical practice (De Casterlé et al., 2008; Deshpande & Joseph, 2009; Goethals et al., 2010). For example, it can be difficult to address bad practices or bad attitudes in someone with whom you work on a daily basis; this was confirmed by several of our participants. In this respect, registering ethical dilemmas digitally may be a safer practice if it leads to the dilemmas being addressed generally in a professional forum such as an ethics reflection group. The caveat is that someone with ethical and methodological competence must lead the ethical discussion (Tønnessen et al., 2016).

Ethical reflection requires both time and space, which the nurses in the interviews considered lacking. Instead, they found that opportunities to meet, vent and discuss were steadily diminishing. Although relatively few of the nurses digitally stated that ethical challenges continued to occupy their thoughts after their shift ended, the interviews showed that the digital reflection was insufficient to cope with moral distress. However, they found the digital registration easy to use and accessible, and envisioned that it would be effective if it was addressed by the manager.

### The Opportunity Space for Digital Ethical Reflection

The study findings indicate that digital ethical reflection can increase nurses’ ethical awareness and give managers important insight into employees’ experiences in the ward. When someone receives the nurses’ reports of their ethical challenges and pays attention to them, an opportunity arises to view the challenges from a different angle and see new perspectives (Koskinen & Lindström, 2015). Digital ethical reflection can provide managers with a starting point for addressing the ethical challenges that nurses face without the nurses feeling uncomfortable or exposed. Some nurses may be reluctant to express their values and beliefs and may perceive the digital format as a safe space. Although the categorization of ethical dilemmas according to the four ethical principles may seem narrow, their use increases awareness and opens further avenues for reflection.

### Limitations and Strengths

This study took place over a short period of time and was conducted in a limited geographical context in two medium-sized Norwegian municipalities, thereby limiting the generalizability of the findings. At the same time, our findings on ethical challenges are recognizable in previous research conducted both nationally and internationally. The small sample size limited our ability to draw statistical conclusions. Additional studies are needed using a larger sample size to further examine the effects of experience, busyness, and type of shift on nurses’ perceived ethical challenges. A weakness of the tool was that each registration only concerned one challenge. Multiple challenges on the same shift required multiple registrations, and the time required may have led to someone failing to report. The questions themselves provided no guidance on how ethical challenges should be understood or to whom they were linked. Obviously, ethical challenges arise in relation to the patient and the care provided. At the same time, ethical challenges can also be experienced in relation to colleagues, relatives, or management, for example. The registration allowed the nurses to describe the “situation” in which the ethical challenge arose, but few actually made use of this.

Another limitation is that no demographic variables were considered. Examining the significance of demographic variables such as age, education, health care experience, and current workplace could have broadened our understanding of ethical challenges. However, our main concern was to investigate whether the tool can act as ethical support for ethics work in long-term care. The fact that managers assisted with recruitment may have influenced what the nurses wanted to register digitally or convey in interviews. At the same time, the findings suggest that nurses want their managers to be more informed about and involved in handling ethical challenges in clinical practice. The findings must nevertheless be considered in light of the risk of underreporting. Another potential limitation is that the participants consisted of only women. Women, admittedly, make up the majority of staff in long-term care, but future studies should include both sexes, as other studies have pointed to gender differences in experiences of moral distress (O’Connell, 2015). This study was developed in close partnership with the field of practice and with clinicians who would benefit from the knowledge gained.

### Implications and Recommendations

The findings constitute an important contribution to further development and testing of the tool on a larger scale, which the research team is working on. The findings also have transfer value for ethics work in general in long-term care because they emphasized the need for tools that can help identify everyday ethics. However, the identification only provides a starting point for further work dealing with ethical challenges. The findings are particularly aimed at nursing managers who should more actively acquire an overview of the challenges nurses experience and take responsibility for making the challenges the subject of reflection in a suitable arena.

## Conclusion

The nurses in this study consider the digital ethical reflection to be practical and easy to complete. Reflecting digitally helped the nurses become more aware of the ethical challenges they faced and their need for ethics support through both dialogue and their manager's attention. However, the utility of digital ethical reflection is partly dependent on the manager reading the reflections and helping the nurses by addressing their ethical challenges in, for example, ethics reflection groups.
